# ASK1 Mediates Apoptosis and Autophagy during oxLDL-CD36 Signaling in Senescent Endothelial Cells

**DOI:** 10.1155/2019/2840437

**Published:** 2019-10-22

**Authors:** KyoungJoo Cho, Seung Ho Choi

**Affiliations:** ^1^Department of Life Science, Kyonggi University, Suwon, Republic of Korea; ^2^Department of Health Sciences and Technology, Sunggyungwan University, Seoul, Republic of Korea; ^3^Samsung Biomedical Research Institute, Research Institute for Future Medicine, Samsung Medical Center, Seoul, Republic of Korea

## Abstract

Vessel damage by oxidized low-density lipoprotein (oxLDL) increases reactive oxygen species (ROS) and the membrane receptor cluster of differentiation 36 (CD36), involving various vascular pathological processes. In this study, the role of apoptosis signal-regulating kinase 1 (ASK1) as a cellular effector via the oxLDL-CD36 signaling axis, and its related mechanism as a downstream responder of CD36, was investigated in senescent human aortic endothelial cells (HAECs). To inhibit oxLDL-triggered vascular damage, HAECs and monocytes were treated with the CD36-neutralizing antibody or the ASK1 inhibitor NQDI-1. The oxLDL-triggered increases in ROS and CD36 elevated active ASK1 in the senescent HAECs. The ROS increase induced apoptosis, whereas CD36 neutralization or ASK1 inhibition protected against cell death. The blocking of CD36 increased senescent HAEC autophagy. In monocytes, oxLDL also induced CD36 expression and autophagy, the latter of which still occurred following ASK1 inhibition but not after CD36 neutralization. These findings suggest that oxLDL exposure activates ASK1, as a CD36 downstream responder, to accelerate apoptosis, particularly in senescent HAECs. ASK1's involvement in monocytic autophagy was due to endoplasmic reticulum stress resulting from the oxLDL load, suggesting that oxLDL loading on aged vessels causes atherosclerotic endothelial dysfunction mediated by active ASK1.

## 1. Introduction

The atherosclerotic process is mediated by dysregulated vessel and blood components, which is the leading cause of cerebro- and cardiovascular disease. Atherosclerotic lesions result from complex inflammatory processes in which monocytes, T cells, and lipoproteins interact with vessels and vessel components [1]. Endothelial dysfunction or activation is one of the main factors of atherosclerosis initiation [2]. In the early atherosclerotic stage, endothelial cell death including apoptosis or autophagy plays a crucial role in atherosclerotic plaque regression or instability [3]. Atherosclerosis and its associated clinical outcomes progress more severely in particularly senescent endothelial cells [4], and numerous senescent endothelial cells are located in the human aorta [5]. As aging progresses, atherosclerotic lesions involved with human atherosclerosis are prone to arise in the human aorta and coronary arteries, which contain senescent endothelial cells [6].

Extracellular and intracellular reactive oxygen species (ROS) are generated during the atherosclerotic process, which is an important leading factor of atherosclerosis development [7]. During oxidation in the vessels, there are changes in their physicochemical properties, such as lipid charge, size, and content. Furthermore, oxidized low-density lipid (oxLDL) becomes different from natural LDL. The components of oxLDL activate endothelial cells, inducing the expression of adhesion molecules such as E-selectin and vascular cell adhesion molecule-1 (VCAM-1) on the endothelial surface of the artery [2]. Since oxLDL can induce vascular ROS production [8], trigger endothelial dysfunction [9], and initiate atherosclerosis progression [10], oxLDL internalization is a critical step in atherosclerosis-related endothelial damage, as well as macrophage foam cell formation [11]. Oxidative stress triggered by vascular cellular ROS stimulates CD36 expression on the surface of various cells, such as vascular endothelial cells, smooth muscle cells, macrophages, and platelets [12]. The scavenger receptor CD36 recognizes oxLDL and mediates its uptake into cells and plays a key role in atherosclerosis pathogenesis. Additionally, CD36 has multiple functions in apoptosis [13], fatty acid transport [14], and angiogenesis inhibition [12]. Previous studies have demonstrated that some kinases, such as mitogen-activated protein (MAP) kinase families, are involved in CD36 signal transduction in monocytes and endothelial cells [13]. oxLDL-induced JNK activation regulates the redox status in endothelial mitochondria; MnSOD is JNK-dependently degraded by ubiquitination; and activation of the JNK pathway leads to endothelial apoptosis [15]. In macrophages exposed to oxLDL, macrophage CD36 was also reported to be linked with MAP kinase, JNK1, and JNK2 [11].

Though the CD36 signaling pathway in atherosclerosis is potentially important, the downstream signaling pathway in endothelial cells is not fully understood. Our study was aimed at investigating the downstream partner molecules responsible for regulation in human endothelial cells and monocytes. Under conditions of vessel damage resulting from oxLDL, this study investigated the redox-sensitizing role of apoptosis signal-regulating kinase 1 (ASK1, MAP3K5) via the oxLDL-CD36 pathway in senescent human endothelial cells. This study identifies a mechanism via which CD36 interacts with ASK1, which acts as a downstream responder of CD36 and is sensitive to oxidative/redox stress.

## 2. Materials and Methods

### 2.1. Cell Culture, Treatment, and Drugs

Human aortic endothelial cells (HAECs) and human THP-1 monocytic cells were used in this study. HAEC cells were purchased from Lonza (Basel, Switzerland) and cultured in endothelial growth media EBM-2 recommended by the manufacturer (Lonza, Switzerland). THP-1 cells were obtained from the American Type Culture Collection (ATCC, Manassas, VA, USA) and cultured in RPMI-1640 (Gibco, Waltham, MA, USA). All cells were maintained at 37°C in a 5% CO_2_ atmosphere. Oxidized LDL (40 *μ*g/mL, Acesar, Waltham, MA, USA) were exposed on each cell for 24 hours to 48 hours. NQDI-1 (ASK1 inhibitor, Tocris Bioscience, Bristol, UK) was dissolved in DMSO and treated on each cell with 600 nM to inhibit ASK1 activation at 4 hours before oxLDL exposure. For the masking receptor, CD36, neutralizing the CD36 antibody (FA6-152, Abcam, Cambridge, MA, USA), was pretreated on each cell (2 *μ*g/mL) 4 hours before oxLDL exposure. To restore ASK1, the peptide for ASK1 was synthesized to contain the active sites (Thr845) of ASK1 from amino acids 836–875.

### 2.2. Cell Viability Assay and Cellular Senescence Activity Assay

THP-1 (1 × 10^5^ cells/mL) and HAEC (5 × 10^4^ cells/mL) cells were seeded in a 96-well microplate. The next day, oxLDL was treated with each concentration (10, 20, 40, and 80 *μ*g/mL) on each well and cells were incubated for 24 hours or 48 hours. After oxLDL treatment, 10 *μ*L of cell counting kit-8 reagent (CCK-8, Dojindo, Japan) was added to each well and cells were incubated for another 3 hours in a CO_2_ incubator. The viability for each cell was measured with the absorbance at the 450 nm wavelength using an ELISA plate reader. Cell viability was expressed as a percentage relative to the control.

In the case of human endothelial cells, we tried to investigate the response under the aging condition and used longer passaged HAECs which were assayed cellular senescence using a senescence-associated *β*-galactosidase (SA-*β*-gal) activity kit (Enzo, Farmingdale, NY, USA). Expression of an acidic SA-*β*-gal is one common biochemical marker of aged cells. Based on the principle, cell lysates on the 96 well plate were added with an assay buffer including fluorescence substrates and were incubated for 3 hours. The activity measured the fluorescence at 360 nm for excitation and 465 nm for emission with LS50B (Perkin Elmer, Norwalk, CT, USA).

### 2.3. Western Blot Analysis

The changes of each protein expression level were confirmed by Western blot analysis. Cells were washed with ice-cold PBS and harvested. Collected cells were lysed in a RIPA lysis buffer for 30 minutes on ice. RIPA was composed with 50 mM Tris·HCl (pH 7.5), 150 mM NaCl, 1% nondiet P-40, 0.1% SDS, and 0.5% sodium deoxycholate and supplemented with protease and phosphatase inhibitors (Protein Halts, Thermo Fisher, Waltham, MA, USA). The equal amounts of protein were loaded and separated on a 4-12% SDS-polyacrylamide gel and transferred onto a polyvinylidene difluoride membrane (PVDF, Millipore, Darmstadt, Germany). After the blocking step, each blot was probed overnight at 4°C with specific primary antibodies as follows: phosphorylation-ASK1 (p-ASK1) (1 : 1000; Cell Signaling Biotechnology, Danvers, MA, USA), ASK1 (1 : 500; SCBT, Santa Cruz, CA, USA), CD36 (1 : 500, Abcam), Beclin (1 : 1000, Cell Signaling Biotechnology), TRAF2 (1 : 500, SCBT), Trx (1 : 500, SCBT), SQSTM1/p62 (1 : 1000, Cell Signaling Biotechnology), CHOP (1 : 1000, Cell Signaling Biotechnology), pJNK (1 : 500, SCBT), p-p38 (1 : 500, SCBT), or LC3II (1 : 1000, Cell Signaling Biotechnology). Each blot was then incubated with HRP-conjugated anti-mouse or anti-rabbit IgG anitibodies (Jackson ImmunoResearch, West Grove, PA, USA) for 1 hour at room temperature. For internal loading control, blots were probed with HRP-conjugated *β*-actin (1 : 2000, SCBT). Bands were detected by chemiluminescence (ECL, Pierce, Dallas, TX, USA) and visualized on LAS 4000 (Fujifilm, Tokyo, Japan). For semiquantification, the density of each band was measured using ImageJ software (NIH).

### 2.4. Flow Cytometry Analysis to Quantify Total ROS or Autophagy

To quantify total ROS of the cells, the ROS-ID® Total ROS/Superoxide detection kit (Enzo, USA) was used and experimental procedures followed the manufacturer's protocol. Cells (HAEC and THP-1) treated with each condition were stained with a detection reagent supplied by the ROS detection kit and analyzed using flow cytometry. General and total oxidative stress levels were monitored in the green channel. For each control, pretreatment with NAC (a general ROS inhibitor) prevented the formation of ROS (a negative control) and treatment with pyocyanin (ROS/NO inducer) induced ROS (a positive control). Cell population excluding cell debris was gated and each data represented each event number as a percentage.

To quantify autophagy in HAEC cells or THP-1 cells, a CYTO-ID Autophagy Detection Kit 2.0 (Enzo, USA) was used and the experiment was performed following the manufacturer's protocol. The kit detected the 488 nm excitable green fluorescence by the reagent supplied in the kit, which is a marker for autolysosomes and earlier autophagic compartments. After treatment under oxLDL, the prepared cells were stained using the Green Detection Reagent 2 in the kit. As a positive control, rapamycin (0.5 *μ*M) and chloroquine (10 *μ*M) were treated on the cells and the cells were additionally cultured for 24 hours to induce autophagy. Autophagic cells were analyzed using flow cytometry, and each autophagic cell was shown in percentage.

### 2.5. Immunocytochemistry

HAECs were plated on the multiwell plate containing a coverslip 24 hours prior to each treatment and oxLDL exposure. After appropriate treatment under oxLDL exposure, HAECs were washed with ice-cold PBS. Cells were fixed in 4% PFA for 20 minutes and permeabilized with 1% Triton X-100 in PBS. Prepared cells were blocked in 3% BSA in PBS for 1 hour and then incubated with each primary antibody overnight at 4°C as follows: anti-rabbit CD36 (1 : 100, Abcam), anti-mouse Trx (1 : 100, SCBT), and anti-rabbit LC3II (1 : 100, Cell Signaling Biotechnology). And then, each sample was incubated with fluorescence-conjugated secondary antibodies for 1 hour at room temperature. We used fluorescein isothiocyanate- (FITC-) conjugated donkey anti-rabbit (1 : 200, Jackson Immunoresearch, West Grove, PA, USA) or rhodamine-conjugated goat anti-mouse (1 : 200, Jackson Immunoresearch). Each sample was washed with PBS three times for 3 min each and then mounted with VECTASHIELD containing 4′,6-diamidino-2-phenylindole (DAPI) (Vector Laboratory, Carlsbad, CA, USA), and images were obtained using a Zeiss LSM 700 confocal microscope (Carl Zeiss, Thornwood, NY, USA). For staining the lysosome, LysoTracker (Molecular Probe, Eugene, OR, USA) was used using the manufacturer's instruction.

### 2.6. Cleaved Caspase-3 Activity Assay with ELISA

Cleaved caspase-3 activities were measured by using an enzyme-linked immunosorbent assay (ELISA) kit (R&D systems, Minneapolis, MN, USA) according the to manufacturer's instructions. The captured antibody was diluted at a working concentration (2 *μ*g/mL) in PBS and coated 100 *μ*L into each well. After blocking the plate with block buffer, each sample and standard (the human/mouse cleaved caspase-3 (Asp175)) were triplicated by adding 100 *μ*L and incubated for 2 hours at room temperature. The plate was washed and streptavidin-HRP was added. Substrate solution was added and read at 450 nm.

### 2.7. Quantitative Real-Time PCR Analysis

Each RNA of HAEC or THP-1 cell was manually extracted using TRIzol (Invitrogen, Waltham, MA, USA). cDNA and real-time PCR were performed with the One Step SYBR PrimeScript RT-PCR (TaKaRa, Japan) kit according to the manufacturer's instructions. Primers for human CD36 and *β*-actin as an internal control are as follows: human CD36 forward, 5′-TCTTTCCTGCAGCCCAATG-3′, reverse, 5′-AGCCTCTGTTCCAACTGATAGTGA-3′; human *β*-actin forward, 5′-GGGRCAGAAGGATTCCTATG-3′, reverse, 5′-GGTCTCAAACATGATCTGGG-3′. ABI StepOne Plus (Applied Biosystems, USA) was used for this quantitative real-time PCR reaction. The step for reverse transcription was done at 42°C for 5 min and at 95°C for 10 sec. The PCR amplification step was performed at 40 cycles of denaturation at 95°C for 5 sec and annealing and extension at 60°C for 60 sec. Each sample was performed in triplicate with no template controls and analyzed.

### 2.8. Statistical Analysis

All data are presented as means ± SD and were analyzed using GraphPad Prism software (version 6.01). Statistical analyses were performed by unpaired *t*-test or one-way ANOVA followed by Bonferroni's multiple comparison test. Differences were considered statistically significant at ^∗^*p* < 0.05, ^∗∗^*p* < 0.01, and ^∗∗∗^*p* < 0.001.

## 3. Results

### 3.1. oxLDL Increases ROS and Induces CD36 in HAECs

**Figure 1:**
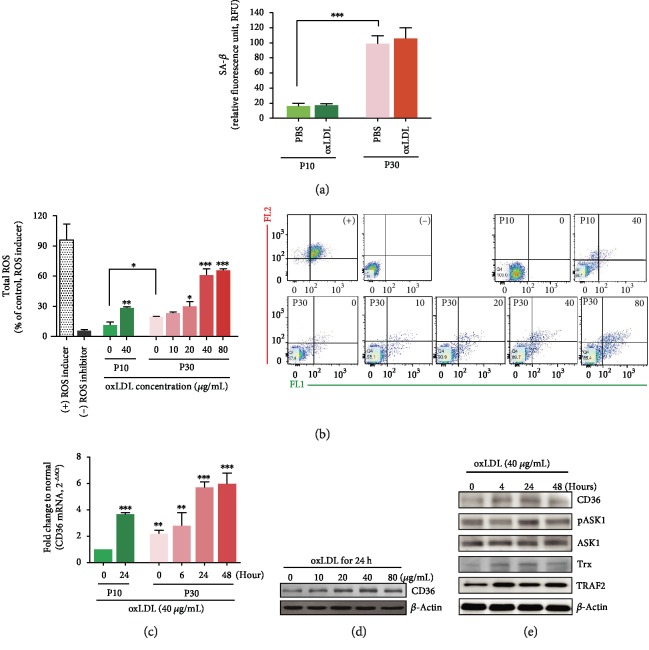
oxLDL induces ROS overexpression and intracellular signals in senescent human aortic endothelial cells (HAECs). (a) SA-*β*-Gal activities in passage 10 (P10) and passage 30 (P30) of HAECs were assayed with or without oxLDL (40 *μ*g/mL) for 24 hours. (b) Total intracellular ROS amounts increased in an oxLDL dose-dependent manner up to a concentration of 40 *μ*g/mL and increased more in senescent HAECs compared to young cells. (c) Stimulation with oxLDL (40 *μ*g/mL) increased the CD36 mRNA level in a time-dependent manner. Each level is shown as the relative fold changes compared to normal young HAECs. (d) The CD36 protein expression rose when HAECs were exposed to oxLDL. (e) The protein expressions of CD36, pASK1, ASK1, Trx, and TRAF2 were analyzed via a time-dependent Western blot at 40 *μ*g/mL oxLDL. The ROS inducer-treated group was presented as a positive control, and the ROS inhibitor-treated group was shown as a negative control. Results are expressed by mean and standard deviation. Significance is presented as follows: ^∗^*p* < 0.05, ^∗∗^*p* < 0.01, and ^∗∗∗^*p* < 0.001.

To investigate the response mechanism under aged vessel conditions, we used HAEC passage 10 (P10) to analyze normal conditions and passage 30 (P30) for senescent conditions. The cell senescence assay confirmed that the P30 HAEC was senescent by analyzing SA-*β*-Gal activity (Figure 1(a)). The viability of the senescent HAECs was evaluated at oxLDL concentrations of 10, 20, 40, and 80 *μ*g/mL for 24 hours (data not shown). At oxLDL concentrations of 40 *μ*g/mL and 80 *μ*g/mL, HAEC viability decreased to 70%, which was statistically significant. oxLDL exposure triggers intracellular ROS production. The total ROS amounts had already been elevated in senescent HAECs and were further increased by oxLDL (at 40 *μ*g/mL for 24 hours). Total oxLDL-induced ROS started to increase from 20 *μ*g/mL oxLDL and escalated at 40 *μ*g/mL in a concentration-dependent manner (Figure 1(b)). This study was performed with senescent HAECs. Endothelial CD36, one of the main receptors of oxLDL, increased 24 hours later due to oxLDL exposure (40 *μ*g/mL). This was demonstrated by CD36 mRNA and presented with a relative fold change to young HAECs (passage 10) (Figure 1(c)). The mRNA level was raised by oxLDL in P10 HAECs, as well as by the senescent condition itself. Furthermore, the amounts of CD36 protein expressed also increased after 24 hours of oxLDL exposure (Figure 1(d)). Each amount that was quantified by Western blot analysis is shown in Supplementary [Supplementary-material supplementary-material-1] (Fig. [Supplementary-material supplementary-material-1]). On the oxLDL-CD36 and ROS signaling axis, changes in protein expression levels were investigated at each exposure time under fixed oxLDL concentration (40 *μ*g/mL). ASK1 is one of the first responders to oxidative stress and is closely involved in endoplasmic reticulum (ER) stress by overloaded intracellular cargo. ASK1 activation peaked at 24 hours, followed by increased TNF receptor-associated factor 2 (TRAF2) for the ER stress response and increased thioredoxin (Trx) for redox sensing (Figure 1(e) and Fig. [Supplementary-material supplementary-material-1]).

### 3.2. oxLDL-CD36-Derived ROS Induces Cell Death via the ASK1 Pathway

**Figure 2:**
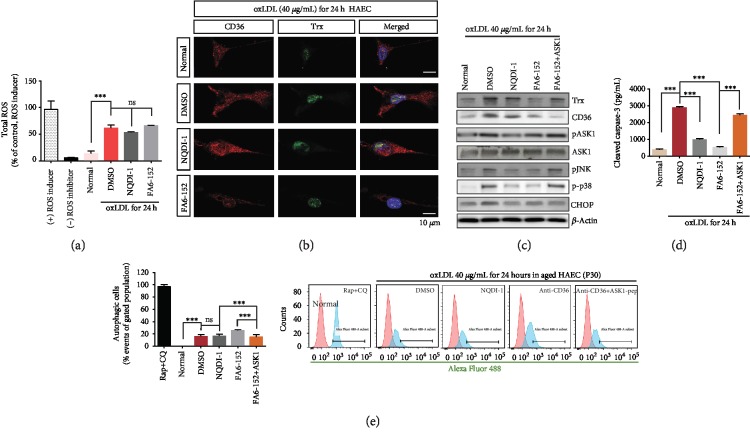
Augmented intracellular ROS derived by oxLDL induces apoptotic cell death rather than autophagy via the MAP kinase pathway in senescent HAECs (P30). (a) The inhibition of ASK1 or blocking of CD36 did not change the total ROS amounts that were increased by oxLDL. (b) CD36 and Trx expression were detected by each antibody in each treated group via immunocytochemistry. (c) Western blot analysis showed the protein level of CD36, Trx, pASK1, pJNK, p-p38, and CHOP. Trx, the redox sensor molecule, was downregulated by blocking CD36 and raised again by adding the ASK1 peptide. (d) Apoptotic cell death was assayed by analyzing cleaved caspase-3 activity via ELISA. Cell death was reduced by inhibiting ASK1 or blocking CD36, but supplemented ASK1 on CD36-masked HAECs caused its reversal. (e) Autophagy assay showed that oxLDL led to autophagy. Autophagy increased by blocking CD36, not by inhibiting ASK1. Normal: senescent HAECs without oxLDL; FA6-152: masking antibody for CD36; NQDI-1: ASK1 inhibitor. Results are expressed by the mean and standard deviation. Significance is presented as follows: ns: not significant. ^∗∗^*p* < 0.01 and ^∗∗∗^*p* < 0.001.

This study was performed at 40 *μ*g/mL oxLDL for 24 hours in senescent HAECs. To regulate the initial harmful changes resulting from ROS increase in senescent HAECs, CD36 was blocked by anti-CD36 antibody (FA6-152) and ASK1 activity was inhibited by NQDI-1, the ASK1 inhibitor. The accumulation of ROS in the senescent HAECs was not much different following treatment with either FA6-152 or NQDI-1 (Figure 2(a) and Fig. [Supplementary-material supplementary-material-1]). Trx is induced by oxLDL-CD36 signaling, and its expression was reduced when oxLDL-CD36 signaling was hindered by masking endothelial CD36 with antibody (Figures 2(b) and 2(c)). However, active ASK1 inhibition failed to decrease the level of Trx, a redox sensor and upstream molecule of ASK1. When CD36 was blocked, endothelial CD36 protein level was scarcely detected and the Trx protein level was also diminished (Figure 2(b)). The ASK1 inhibitor, NQDI-1, clearly inhibited active ASK1 (pASK1-Thr845), but did not inhibit Trx. Blocking CD36 also reduced active ASK1 but not as much as NQDI-1 (Figure 2(c) and Fig. [Supplementary-material supplementary-material-1]). Inhibition of active ASK1 concomitantly and correspondingly reduced pJNK and p-p38, resulting in reduced HAEC death, as indicated by the cleaved caspase-3 assessment (Figure 2(d)). oxLDL-derived ER stress was shown to increase the CHOP protein. This study investigated the role of ASK1 as the intracellular responder of the oxLDL-CD36 signaling axis in senescent HAECs. When CD36 was masked, the ASK1 peptide was introduced into HAECs. Under these conditions, supplemented ASK1 reciprocally increased pASK1, whereas it did not affect the protein level of CD36 and CHOP (Figure 2(c) and Fig. [Supplementary-material supplementary-material-1]). Apoptotic endothelial cell death by oxLDL was protected against by both CD36 blocking (FA6-152) and ASK1 inhibition. However, the ASK1 peptide supplement exacerbated the apoptotic cell death of senescent HAECs (Figure 2(d)). The loading of oxLDL onto HAECs also led to autophagy and CD36 masking, triggering further autophagy (Figure 2(e)).

### 3.3. oxLDL Induces Autophagy in THP-1 Cells

**Figure 3:**
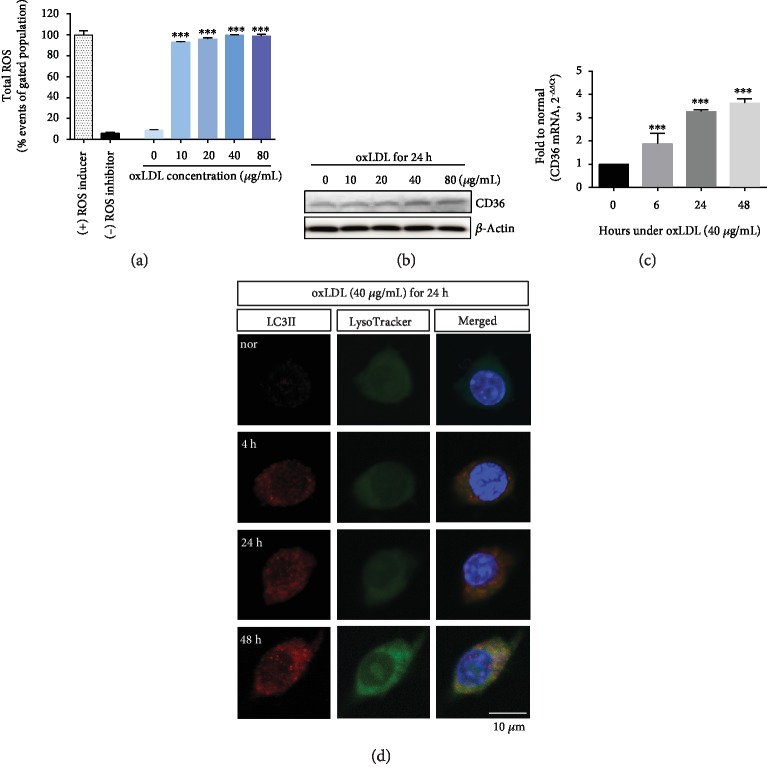
oxLDL-stimulated human monocytes (THP-1 cells) had increased CD36 and induced autophagy. (a) Total ROS amounts significantly increased from an oxLDL concentration of 10 *μ*g/mL. (b) Western blot analysis demonstrated that CD36 protein expression was enhanced to levels as high as oxLDL concentration in THP-1 cells. (c) CD36 mRNA levels were elevated in a time-dependent manner at an oxLDL concentration of 40 *μ*g/mL, as demonstrated by using real-time PCR. (d) Immunocytochemistry demonstrated that LC3II-positive THP-1 cells were detected at 40 *μ*g/mL oxLDL in a time-dependent manner. At 48 hours, LysoTracker was fully shown as being colocalized to LC3II. Results are expressed by the mean and standard deviation. Significance is presented as follows: ^∗∗∗^*p* < 0.001.

Atherosclerosis results from the combination of endothelial cells with blood factors such as monocytes. This study used human monocytes and human aortic endothelial cells. Under oxLDL exposure, total ROS amounts in THP-1 cells increased from the concentration of oxLDL 10 *μ*g/mL (Figure 3(a)). CD36 protein levels increased with oxLDL concentration (Figure 3(b) and Fig. [Supplementary-material supplementary-material-1]). Additionally, CD36 mRNA was raised in a time-dependent manner at an oxLDL concentration of 40 *μ*g/mL (Figure 3(c)). These changing patterns were similar to the results for HAECs. Immunocytochemistry showed that THP-1 cells exposed to oxLDL (40 *μ*g/mL) had increased autophagy, as detected by LC3II-positive cells (Figure 3(d)).

### 3.4. ASK1 Mediates Autophagy by Responding to TRAF2 rather than Trx in oxLDL-CD36 Signaling in THP-1 Cells

**Figure 4:**
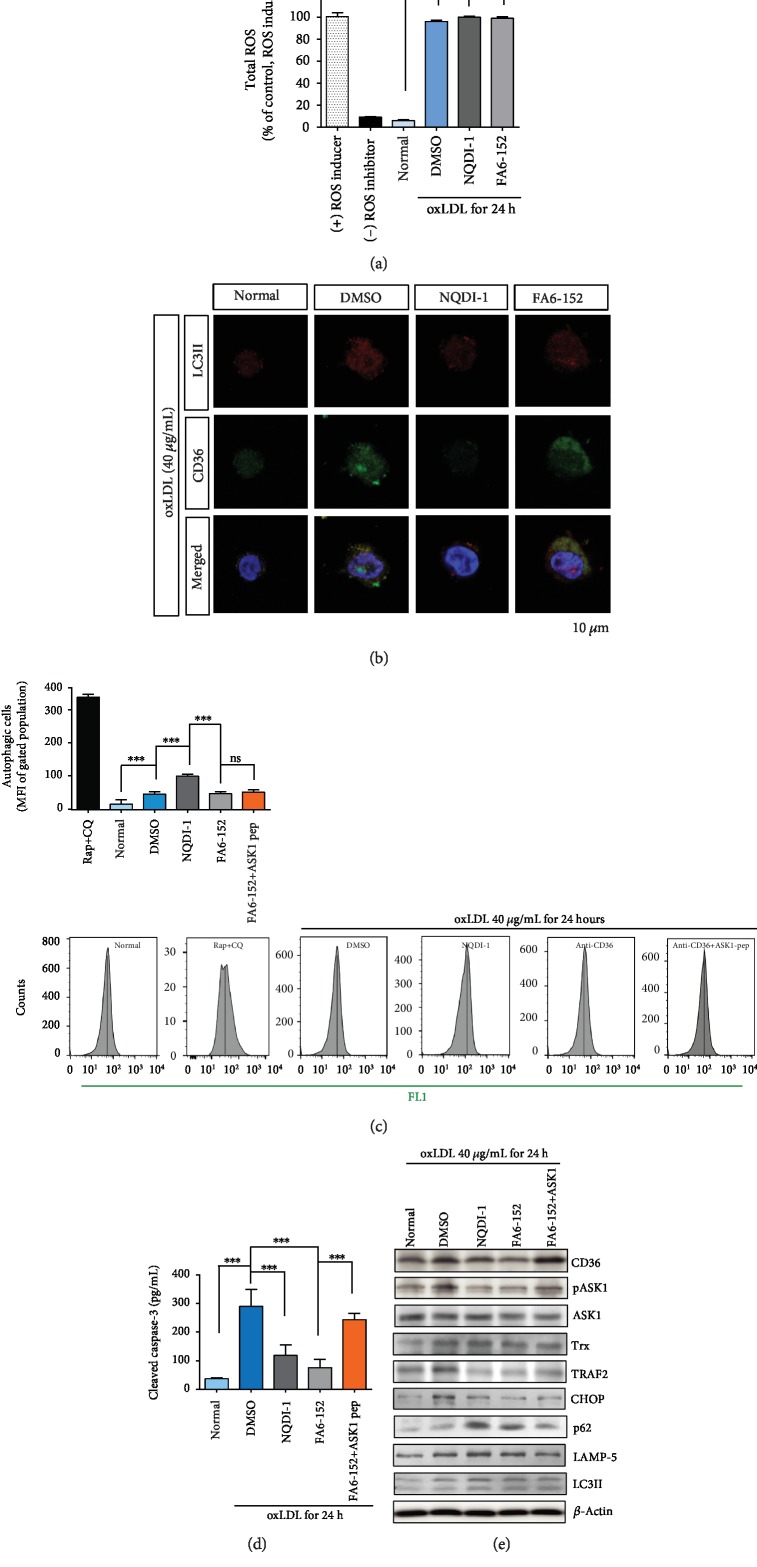
ASK1 modulates autophagy by oxLDL-CD36 signaling in THP-1 cells. (a) Total amounts of ROS in THP-1 cells were not significantly changed by inhibiting ASK1 or by blocking CD36. (b) LC3II and LysoTracker were detected in THP-1 cells and increased in a time-dependent manner. In immunocytochemistry, CD36 and LC3II signals were diminished by inhibiting ASK1 and blocking CD36. (c) Autophagy assay showed that oxLDL led to autophagy, and autophagy increased by inhibiting ASK1, not by blocking CD36. (d) Apoptotic cell death was evaluated by observing cleaved caspase-3 activity. Activity was reduced by inhibiting ASK1 or by blocking CD36, but supplemented ASK1 in CD36-masked THP-1 cells increased cell death. (e) Western blot evaluated the changes in each protein level by blocking CD36 or by inhibiting ASK1. Normal: THP-1 without oxLDL; FA6-152: masking antibody for CD36; NQDI-1: ASK1 inhibitor. Results are expressed by the mean and standard deviation. Significance is presented as follows: ^∗∗∗^*p* < 0.001.

Similar to the results for HAECs, total ROS amounts in THP-1 cells were not significantly changed by CD36 blocking or by ASK1 inhibition (Figure 4(a)). oxLDL induced both CD36-positive and LC3II-positive THP-1 cells (Figure 4(b)). In parallel, CD36-positive and LC3II-positive signals were observed in the THP-1 cells in the CD36-neutralized group. However, in the ASK1-inhibited group, both the CD36-positive and LC3II-positive signals were barely detected in these monocytic cells (Figure 4(b)).

Autophagy was increased in THP-1 cells by inhibiting ASK1 rather than by blocking CD36 (Figure 4(c)), which differed from the results for HAECs (Figure 2(e)). Apoptotic cell death assessed by cleaved caspase-3 was protected against by both CD36 blocking (FA6-152) and by ASK1 inhibition (Figure 4(d)). As with the results for HAECs, adding the ASK1 peptide induced apoptotic cell death. When ASK1 was inhibited, the ER stress-mediating protein TRAF2 was effectively regulated and decreased CHOP as a result. Finally, autophagic signaling molecules such as LAMP-5 and LC3II were reduced by inhibiting ASK1 (Figure 4(e) and Fig. [Supplementary-material supplementary-material-1]). The protein levels of CD36 and Trx were not regulated by ASK1 inhibition in THP-1 cells. This may be because CD36 and Trx are upstream molecules of ASK1 in the oxLDL-CD36 pathway. Blocking CD36 reduced the amounts of Trx, pASK1, CHOP, p62, and LC3II as well as CD36 (Figure 4(e)). In addition, supplementing ASK1 peptide returned each protein level to the damaged level.

## 4. Discussion

The current study showed that (i) oxLDL exposure promoted endothelial and monocytic CD36 signaling, inducing downstream ASK1 activation and subsequent apoptosis or autophagy; (ii) ASK1 modulated the apoptosis of senescent HAECs via Trx as a result of the oxLDL exposure; and (iii) the involvement of ASK1 in monocytic autophagy occurred via TRAF2 as a result of the oxLDL load.

This study used senescent HAECs because senescent endothelial cell accumulation in the aorta of elderly people causes their susceptibility to atherosclerosis (Figures 1 and 2). Cell senescence irreversibly alters both the ability of cells to divide and the expression of cell cycle regulators. Cells that are at senescence become morphologically flatter and longer [16], with endothelial senescence describing a more proinflammatory and proapoptotic state [17]. These senescent endothelial cells are vulnerable to atherogenesis and atherosclerotic progression, as they enhance the recruitment of blood monocytes to the vessel wall [18]. The disturbance of blood flow was shown to promote endothelial cell senescence in atherosclerotic mice, particularly in the ascending aorta and aortic arch [19]. The mechanical stress in the aortic arch can produce more ROS in HAECs under laminar shear stress [20]. oxLDL-triggered ROS production is one of the initiating factors of atherosclerosis progression. The oxidative stress caused by the overgenerated ROS controls the MAP kinase pathway. ASK1, an MAP3K family member, is an earlier cellular signaling responder to ROS [21], ER stress [22], and lipopolysaccharide [23]. It is also involved in cellular differentiation [24], inflammation [25], apoptosis [26], and autophagy [23]. This study focused on the role of ASK1 as a cellular responder and effector via the oxLDL-CD36 signaling axis. The relatively high ROS levels in senescent HAECs were further elevated by oxLDL under atherosclerotic conditions, and the CD36 and pASK1 expression levels were increased as well (Figure 1).

Trx is one of the key regulators of the intracellular redox state and is crucial in atherogenesis [27]. In resting cells, the reduced form of Trx exists as a complex with ASK1, inhibiting the latter's activity. When Trx is oxidized in the presence of ROS, it releases ASK1, which can then be activated by phosphorylation at threonine 845 [21]. Despite the presence of ROS, Trx was reduced by the blocking of CD36 (Figure 2), which could be speculated as being a result of a disconnection within the oxLDL-CD36 signaling axis under oxLDL exposure. Although ASK1 activation in the presence of oxLDL induced apoptosis, it was not enough to fully induce autophagy (Figure 2). ASK1 inhibition effectively defended the senescent HAECs against apoptosis, and the addition of the ASK1 peptide did not lead to any autophagic changes in the cells (Figure 2). Although the physiological role of endothelial autophagy remains poorly understood, it has been shown that the anti-inflammatory effects of autophagy were augmented by mTOR inhibitors in cultured endothelial cells, and the same effect of the mTOR inhibitor in human studies was reported [28]. Our study demonstrated a link between oxLDL and autophagy in senescent HAECs, in that the oxLDL-CD36 signaling axis mediates apoptosis rather than autophagy via the ASK1 pathway. This corresponded with previous reports stating that activated ASK1 increased the p38 MAP kinase activity, particularly in aged mice [29].

In common atherosclerosis, oxLDL exposure raises the ROS levels in human monocytic THP-1 cells and the total ROS amounts were at a similar level in the positive control group (Figure 3). oxLDL accumulation triggers autophagy (Figure 3), which has been regarded as an important mechanism for regulating excess and exogenous lipids [30]. The prolonged vessel damage caused by cholesterol or oxLDL induces ER stress, which can ultimately lead to macrophage death in atherosclerotic plaques [31, 32]. It has been reported that lessening the ER stress could alleviate atherosclerosis, particularly in the advanced stages of atherosclerotic plaque formation in human or mouse macrophages [31, 32]. Prolonged ER stress can induce autophagy as well as apoptosis through the ER-resident protein (IRE1) or downstream effectors (CHOP) [33]. ER stress and autophagy have some common features, inducing either cell death or protection by altering their functions [34]. TRAF2 is an adaptor protein, an intracellular signaling mediator, and an E3 ubiquitin ligase [35]. Upon ER stress, endoplasmic reticulum to nucleus signaling 1 (ERN1) recruits the TRAF2-ASK1 complex [36, 37] and activates downstream kinases. ASK1 inhibition diminished TRAF2 and concomitantly reduced the CHOP and LC3II protein levels (Figure 4). ASK1 inhibition increased THP-1 cell autophagy, but concomitantly, ASK1 inhibition protected THP-1 cells from apoptosis (Figure 4).

## 5. Conclusions

**Figure 5:**
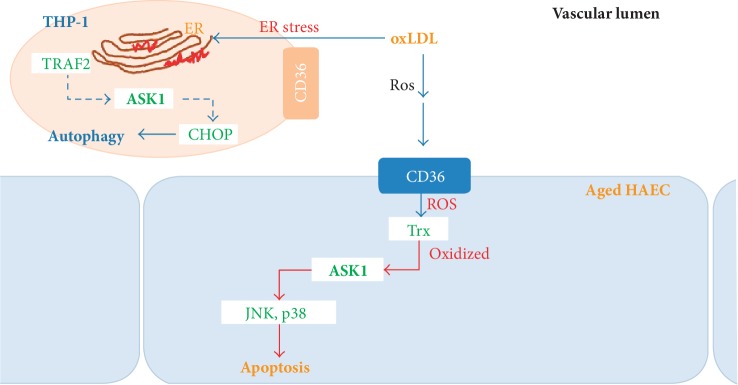
Schematic model depicting the different roles of ASK1 under atherosclerotic conditions. Human endothelial cell or monocyte ASK1 mediates apoptosis or autophagy by oxLDL-CD36 signaling.

This study indicates that the oxLDL-triggered increase in ROS production modulates the expression of both endothelial CD36 and active ASK1. The ASK1 of HAECs or monocytes likely mediates apoptosis or autophagy via oxLDL-CD36 signaling through its different roles under atherosclerotic conditions. This suggests that oxLDL loading on aged vessels can lead to pASK1-mediated atherosclerotic endothelial dysfunction, as depicted in the schematic summary in Figure 5. This may accelerate the development of atherosclerosis in aging individuals.

## Data Availability

The data used to support the findings of this study are included within the article, and the data are as follows: cell viability assay, cellular senescence activity assay, Western blot analysis, ROS assay, autophagy assay, cleaved caspase-3 activity assay, immunocytochemistry, and quantitative real-time PCR.

## Supplementary Materials

Supplementary Materials Supplementary Figure 1: quantified graphs for Western blot analysis. The optical density of each band was assessed by ImageJ software. Each graph demonstrates for Figure 1(d) (A), Figure 1(e) (B), Figure 2(c) (C), Figure 3(b) (D), and Figure 4(e) (E). Significance is presented as ^∗^*p* < 0.05, ^∗∗^*p* < 0.01, and ^∗∗∗^*p* < 0.001. Supplementary Figure 2: dot plot of FACS analysis for total ROS. The percentage of events was presented in each figure, and the events were combined from three quadrants except for the lower left quadrant. Each FACS result demonstrates for Figure 2(a) (A), Figure 3(a) (B), and Figure 4(a) (C).
