# Supplementary material for: ASK1 Mediates Apoptosis and Autophagy during oxLDL-CD36 Signaling in Senescent Endothelial Cells
Source: Oxid Med Cell Longev. 2019 Oct 22;2019:2840437. doi: 10.1155/2019/2840437 (PMC6854215; doi:10.1155/2019/2840437)
Supplement: Supplementary Materials — Supplementary Figure 1: quantified graphs for Western blot analysis. The optical density of each band was assessed by ImageJ software. Each graph demonstrates for Figure 1(d) (A), Figure 1(e) (B), Figure 2(c) (C), Figure 3(b) (D), and Figure 4(e) (E). Significance is presented as ∗p < 0.05, ∗∗p < 0.01, and ∗∗∗p < 0.001. Supplementary Figure 2: dot plot of FACS analysis for total ROS. The percentage of events was presented in each figure, and the events were combined from three quadrants except for the lower left quadrant. Each FACS result demonstrates for Figure 2(a) (A), Figure 3(a) (B), and Figure 4(a) (C). [file 2840437.f1.pdf]

Supplementary Figure 1

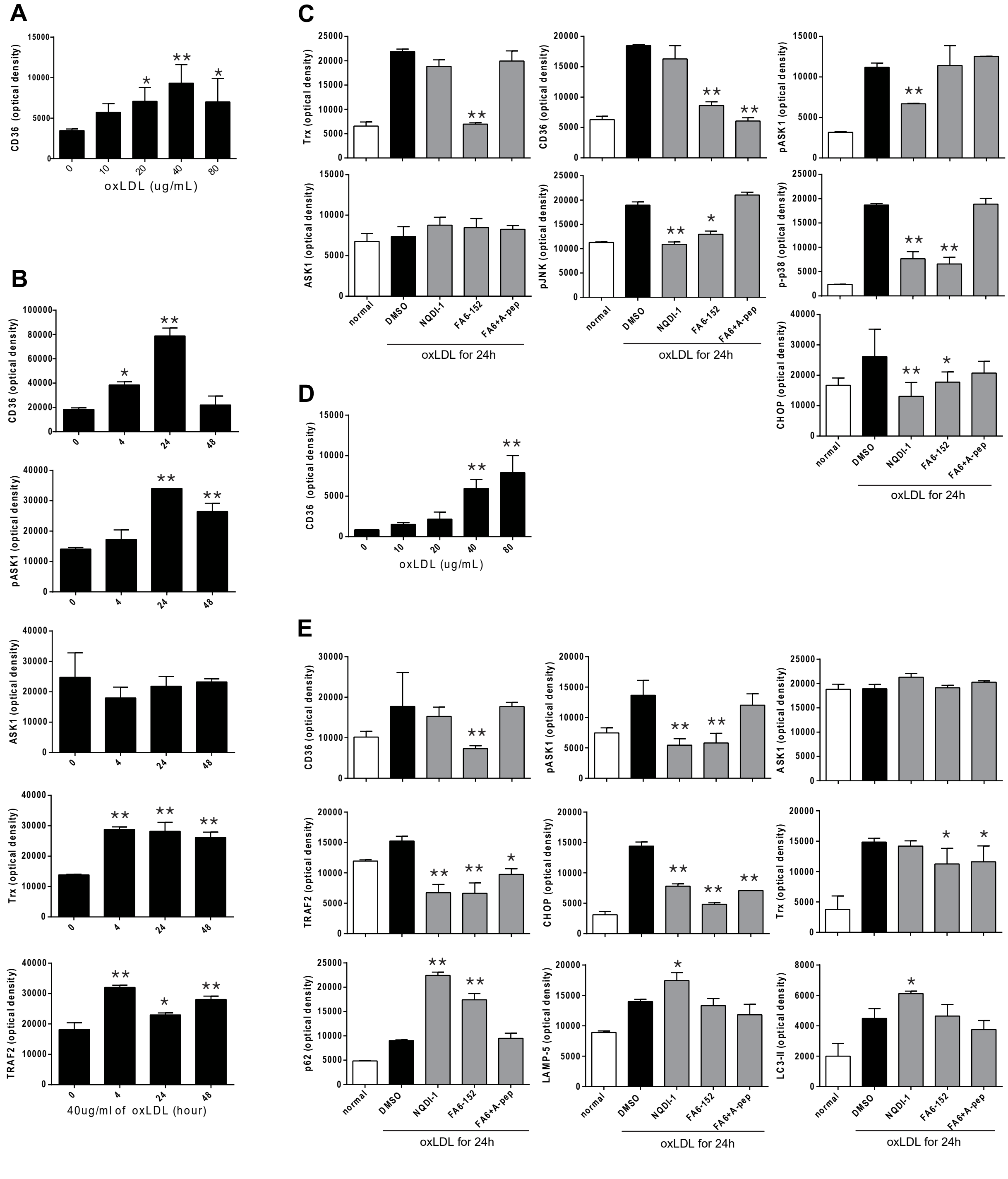

Supplementary Figure 1. Quantified graphs for Westen blot analysis.
Optical density of each band were assessed by ImagJ software. Each graph demonstrates for Figure 1D (A), Figure 1E (B), Figure 2C (C), Figure 3B (D), and Figure 4E (E), respectively. Significance is presented as *p<0.05, **p<0.01, and ***p<0.001

Supplementary Figure S2

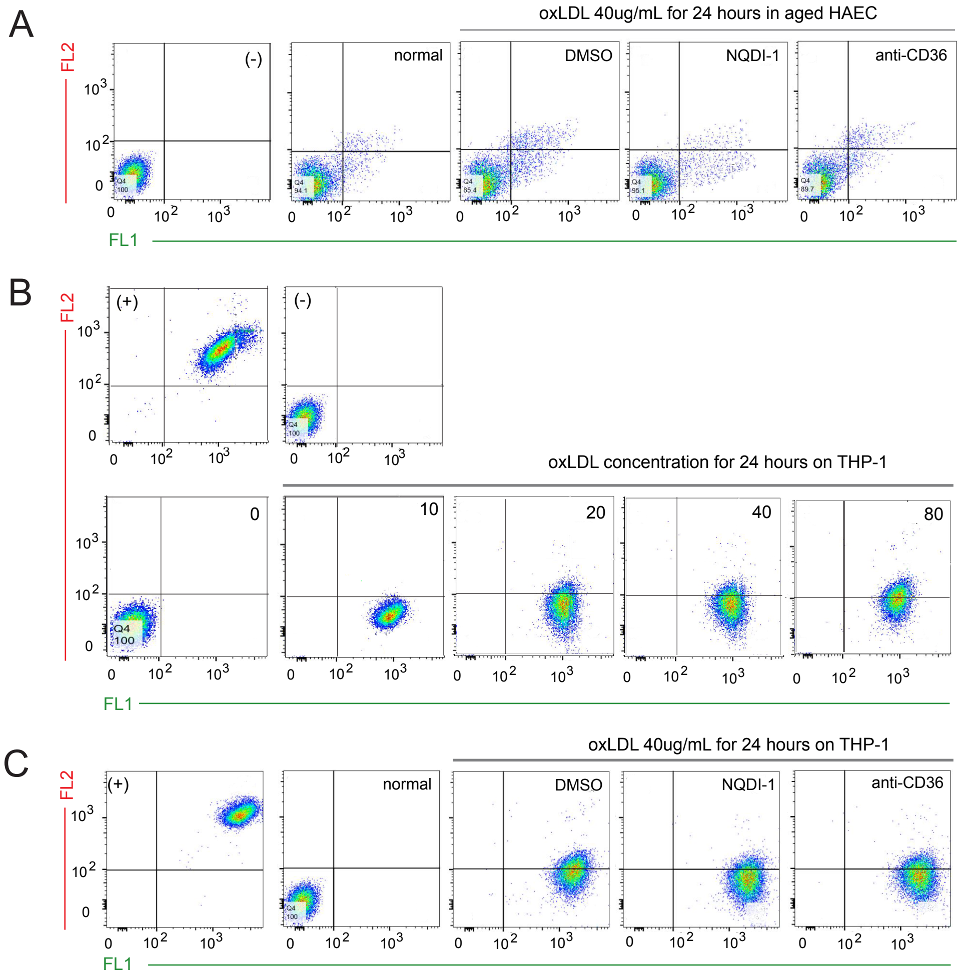

Supplementary Figure 2. Dot plot of FACS analysis for total ROS. The percentage of events were presented in each Figure and the events were combined from three quadrants except of lower left quadrant. Each FACS result demonstrates for Figure 2A (A), Figure 3A (B), and Figure 4A (C), respectively.
